# The relationship between serum NT-proBNP levels and severity of coronary artery disease assessed by SYNTAX score in patients with acute myocardial infarction

**DOI:** 10.3906/sag-1902-26

**Published:** 2019-10-24

**Authors:** Taner SARAK, Muhammed KARADENİZ

**Affiliations:** 1 Department of Cardiology, Faculty of Medicine, Kırıkkale University, Kırıkkale Turkey

**Keywords:** Myocardial infarction, NT-proBNP, severity of coronary artery disease

## Abstract

**Background/aim:**

In the present study, we aimed to investigate the relationship between NT-proBNP and SYNTAX score, which is a measure of the complexity of coronary artery disease.

**Materials and methods:**

We enrolled 405 consecutive patients with myocardial infarction who underwent coronary angiographic examination. Patients were divided into 3 groups according to their SYNTAX scores. Those with SYNTAX score ≤22 were included in the low SYNTAX score group (LSTX), those with a score of 23–32 were included in the intermediate SYNTAX score group (ISTX), and those with a score of ≥33 were included in the high SYNTAX score group (HSTX).

**Results:**

NT-proBNP levels were found to be significantly higher in the HSTX group compared to the other groups (P < 0.001) and in the ISTX group compared to the LSTX group (P < 0.001). The NT-proBNP levels demonstrated an increase from low SYNTAX score to high SYNTAX score tertiles.

**Conclusions:**

NT-ProBNP levels in patients with myocardial infarction on admission were independently associated with extent, severity, and complexity of coronary atherosclerosis as assessed by SYNTAX score.

## 1. Introduction

Coronary artery disease (CAD) remains the most important cause of death in the world. Acute coronary syndromes (ACS) that involve ST elevation myocardial infarction (STEMI), non-ST elevation myocardial infarction (NSTEMI), and unstable angina pectoris (UAP) are important life-threatening disease groups. Various scoring systems are used for risk identification in ACS. One of these systems is the SYNTAX (STX) score, used to estimate the extent and complexity of CAD [1] and consequently early and late results [2]. This scoring system divides patients into different risk groups according to angiographic characteristics and provides foresight into the success of revascularization and early/late prognosis according to risk groups [3–5]. Previous studies have shown that high levels of hs-troponin [6], low-density lipoprotein (LDL), ApoB [7], procalcitonin [8], uric acid [9], and high-sensitivity C-reactive protein (hs-CRP) [10] are associated with high STX scores. N-terminal probrain natriuretic peptide (NT-proBNP) is a biomarker released from the myocardium due to increased myocardial stress and used for diagnosis of heart failure [11]. It has been shown that NT-proBNP was secreted by ischemic myocardium [12] and high levels of NT-proBNP are associated with poor prognosis in myocardial infarction (MI) [13,14] in patients who underwent percutaneous coronary interventions [15]. Thus, high NT-proBNP levels in patients with MI can identify patients who are at a higher risk for adverse cardiovascular events and death. However, it is not clear which mechanism plays a role in the association of high NT-proBNP levels and increased mortality and poor prognosis. The extent and severity of CAD could represent a mechanism to explain this relation.

In the present study, we aimed to investigate the relationship between NT-proBNP and SYNTAX score, which is a measure of the prevalence and complexity of CAD in patients with MI.

## 2. Materials and methods

### 2.1. Ethics statement

This study was approved by an institutional ethical review committee of the Faculty of Medicine, Kırıkkale University, and the reporting of the study conforms to the STROBE statement, along with references to the STROBE statement and the broader EQUATOR guidelines [16]. The study was conducted according to the principles expressed in the Declaration of Helsinki.

### 2.2. Study population

Four hundred and five consecutive patients with MI who underwent coronary angiographic examination between January 2016 and June 2018 were enrolled in the study retrospectively. Patients were classified into two groups according to electrocardiographic findings as NSTEMI or STEMI.

### 2.3. Diagnostic criteria

Patients with typical chest pain for at least 20 min and elevated troponin I values with ≥1 mm ST segment elevation in 2 adjacent leads (>0.2 mV in leads V1, V2, or V3), or a new left bundle branch block in ECG were defined as having STEMI. Patients with typical chest pain for at least 20 min and elevated troponin I values with or without new horizontal ≥0.05 mV ST depression in at least 2 adjacent leads were accepted as having NSTEMI. 

### 2.4. Exclusion criteria

Patients who had no NT-proBNP measurements or had normal troponin I values were not included in the study population. Patients with prior CABG, active infection, chronic inflammatory disease, hepatic and renal disease, known congestive heart failure or cancer, severe valve disease, preprocedural resuscitation, or systolic and diastolic heart failure with Killip class III–IV were excluded. Fifty-four patients were excluded from the study based on the exclusion criteria. 

### 2.5. Laboratory assays and imaging modalities 

Blood samples were collected at admission for NT-proBNP and other routine blood tests. NT-proBNP measurements were made with a COBAS 411 Roche device. Transthoracic echocardiography was performed for all patients within 24 h after hospitalization. Coronary angiography was performed with a General Electric Optima device. 

### 2.6. STX classifications

Coronary artery lesions were evaluated by two invasive cardiologists. STX scores were determined for each coronary lesion with stenosis of >50%, in vessels >1.5 mm, and calculated using the SYNTAX score website (www.syntaxscore.com). Patients were divided into 3 groups according to their SYNTAX scores. Those with a SYNTAX score of ≤22 were included in the low SYNTAX score group (LSTX), those with a score of 23–32 were included in the intermediate SYNTAX score group (ISTX), and those with a score of ≥33 were included in the high SYNTAX score group (HSTX).

### 2.7. Statistical analysis

All analyses were performed using SPSS 21.0 for Windows (IBM Corp., Armonk, NY, USA). Continuous variables were expressed as mean ± standard deviation for parametric variables and as median and interquartile range for nonparametric variables. Categorical variables were represented as numbers and percentages. The Kolmogorov–Smirnov test was used to assess the normality of the distribution; data were analyzed for homogeneity using Levene tests. Comparisons of parametric values between groups were performed by the one-way ANOVA test and nonparametric values were performed by the Kruskal–Wallis test. Categorical variables were compared using the chi-square test. Receiver operating characteristic (ROC) curve analysis was carried out in order to determine the best cut-off value of NT-proBNP; the sensitivity and specificity at that point were obtained. The area under the curve (AUC) was used to determine how NT-proBNP can distinguish between HSTX and other STX groups. Univariate logistic regression analysis was used to identify independent predictors of HSTX. After univariate analysis, significantly obtained variables were used in multivariate logistic regression analysis. Statistical significance was accepted at P < 0.05.

## 3. Results

**Figure 1:**
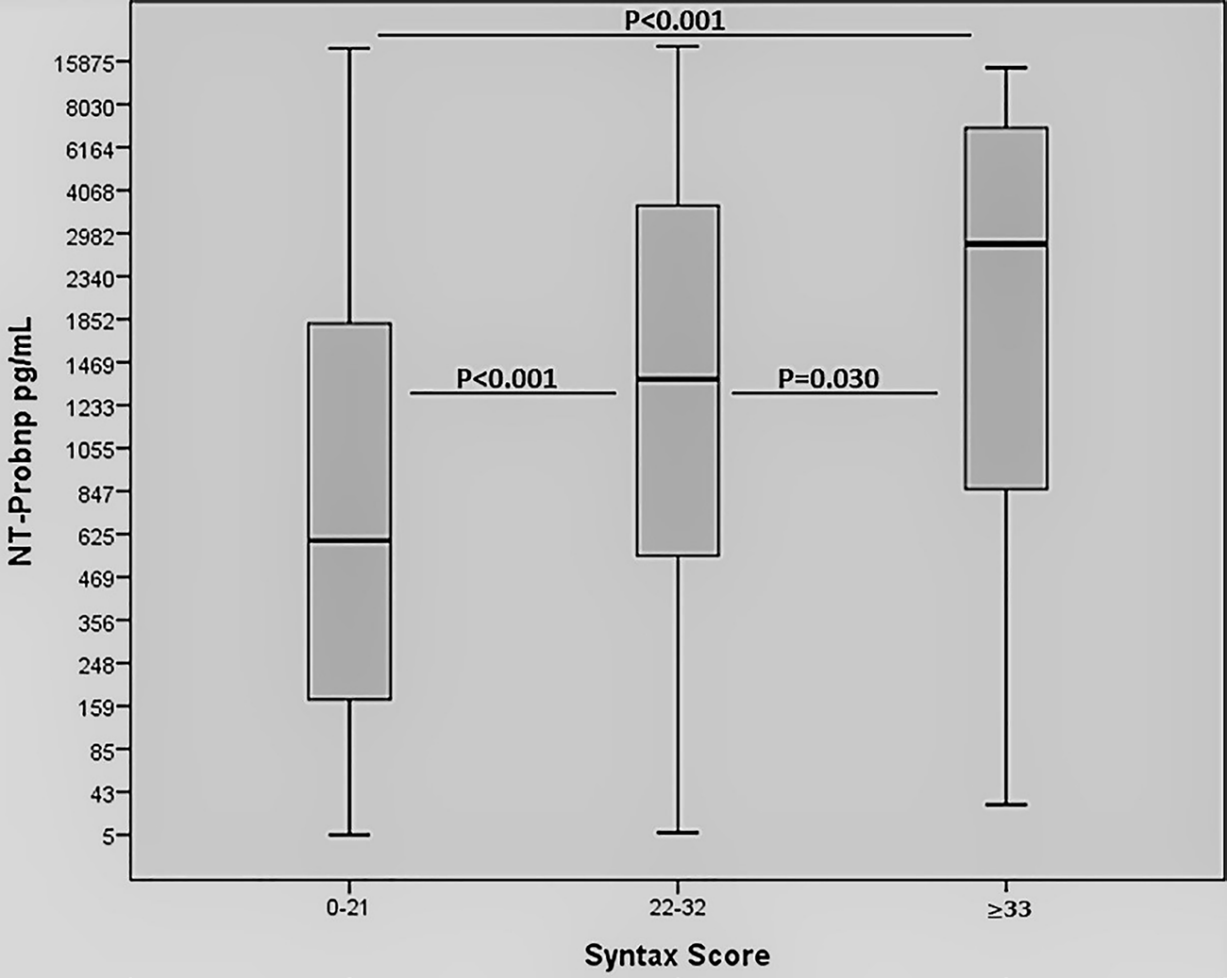
Comparison of N-terminal probrain natriuretic peptide (NT-proBNP) levels among SYNTAX score (STX) groups.

Four hundred and five consecutive patients with MI were included in the study. The baseline characteristics of the study population classified according to SYNTAX score tertiles are shown in Table 1. Mean age was significantly lower in the LSTX group than in the other groups ( P1 < 0.002, P2 < 0.001). Incidence of diabetes mellitus was found to be higher in the HSTX group than in the other groups (P1 = 0.17, P2 = 0.001). Family history and smoking was higher in the LSTX group than in the other groups (P1 = 0.19, P2 = 0.039 and P1 = 0.039, P2 = 0.062, respectively). The ejection fraction was found to be higher in the LSTX group than the other groups (P1 < 0.001, P2 < 0.001). There was no significant difference in the sex, presence of hypertension, hyperlipidemia, prior stroke, blood pressure, or subgroup of MI. Laboratory measurements of the patients stratified into STX tertiles are shown in Table 2. Hemoglobin values ​​were significantly lower in the ISTX group than in the LSTX group (P1 = 0.041), and creatinine clearance levels were significantly lower in the HSTX group than the LSTX group and in the ISTX group than the LSTX group (P1 = 0.020, P2 < 0.001). Serum glucose (P1 = 0.10, P2 = 0.003), HbA1c (P1 = 0.31, P2= 0.048), and troponin levels (P1 < 0.001, P2 = 0.049) were significantly lower in the LSTX group than in the other groups. We found that there was a positive correlation between NT-proBNP and troponin I when we performed Spearman correlation analysis (P = 0.001, r = 0.292). NT-proBNP levels were found to be significantly higher in the HSTX group than in the LSTX group (P < 0.001) and in the ISTX group higher than in the LSTX group (P < 0.001) (Figure 1). The NT-proBNP levels demonstrated an increase from LSTX to HSTX tertiles (Table 2).

**Table 1:** Baseline characteristics of patients classified into SYNTAX score groups.

Variables	LSTX (n = 261)	ISTX (n = 107)	HSTX (n = 37)	P_1_ value	P_2_ value
Age, years (mean ± SD)	60 ± 12	65 ± 13	70 ± 11	0.002	<0.001
Male sex, n (%)	184 (70.2)	62 (57.9)	23 (63.9)	0.081	0.98
Hypertension, n (%)	113 (44.7)	55 (52.9)	21 (61.8)	0.37	0.13
Diabetes mellitus, n (%)	75 (29.5)	41 (39.4)	21 (61.8)	0.17	<0.001
Hyperlipidemia, n (%)	75 (29.6)	31 (29.8)	6 (17.6)	0.99	0.40
Current smoker, n (%)	125 (48.6)	36 (34.0)	10 (27.8)	0.039	0.062
Family history, n (%)	51 (20.2)	13 (12.5)	1 (2.9)	0.19	0.039
Prior stroke, n (%)	4 (1.6)	3 (2.8)	2 (5.6)	0.70	0.83
Left ventricle EF (%)	48 ± 9	41 ± 9	41 ± 10	< 0.001	<0.001
Systolic BP, mmHg	132 ± 26	126 ± 26	125 ± 25	0.15	0.21
Diastolic BP, mmHg	79 ± 14	77 ± 16	74 ± 12	0.42	0.22
STEMI, n (%)	165 (63.0)	71 (66.4)	18 (50.0)		
Non-STEMI, n (%)	97 (37.0)	36 (33.6)	18 (50)		

EF, Ejection fraction; BP, blood pressure; STEMI, ST-segment elevation myocardial infarction. P_1_, Comparison between LSTX and ISTX; P_2_, comparison between LSTX and HSTX.

**Table 2:** Laboratory measurements of patients classified into SYNTAX score groups.

Variables	LSTX	ISTX	HSTX	P_1_ value	P_2_ value
Hemoglobin (g/dL)	14.10 ± 1.8	13.60 ± 2.0	13.40 ± 1.60	0.041	0.34
WBC (103/µL)	10.6 ± 3.2	11.7 ± 3.8	9.9 ± 3.9	0.030	0.31
Platelet (103/µL)	239 ± 71	240 ± 65	232 ± 92	0.98	0.31
Serum glucose (mg/dL)	145 ± 74	162 ± 84	191 ± 93	0.10	0.003
HbA1c (%)	6.8 ± 1.9	7.2 ± 2.1	7.7 ± 2.0	0.31	0.048
eGFR (mL/min)	73 ± 20	67 ± 20	56 ± 15	0.020	<0.001
Total cholesterol (mg/dL)	197 ± 53	193 ± 55	185 ± 47	0.72	0.33
Triglyceride (mg/dL)	135 (91–214)	133 (89–199)	137 (111–223)	0.89	0.43
LDL cholesterol (mg/dL)	125.87 ± 41.12	121.65 ± 49.27	111.53 ± 33.50	0.70	0.13
HDL cholesterol (mg/dL)	40.34 ± 9.17	41.44 ± 10.77	42.42 ± 8.32	0.92	0.85
Peak troponin T (ng/L)	1840 (302–2307)	3262 (603–6102)	3315 (515–5010)	<0.001	0.049
Hs-CRP (mg/dL)	6.8 ± 3.9	7.5 ± 3.7	8.0 ± 3.4	0.38	0.59
NT-ProBNP (pg/mL)	607 (160–1899)	1369 (583–3627)	2760 (779–6865)	<0.001	<0.001

WBC, White blood cell count; HbA1c, glycated hemoglobin; eGFR, glomerular filtration rate; LDL, low-density lipoprotein; HDL, high-density lipoprotein; hs-CRP, high-sensitivity C-reactive protein; NT-proBNP, N-terminal probrain natriuretic peptide. P_1_, Comparison between LSTX and ISTX; P_2_, comparison between LSTX and HSTX.

When evaluated in terms of culprit artery, we found that the mean NT-proBNP values were 2673 pg/mL in left anterior descending artery (LAD) lesions, 2408 pg/mL in circumflex artery (CX) lesions, and 1664 pg/mL in right coronary artery (RCA) lesions. There was a statistically significant difference in NT-proBNP values between LAD and RCA lesions (P = 0.018). However, no significant difference was found between LAD-CX and CX-RCA lesions (P = 0.564 and P = 0.149, respectively).

The presence of multivessel disease and chronic total occlusion, as well as type of treatment between groups, are presented in Table 3. Patients with HSTX had a significantly higher rate of multivessel disease and chronic total occlusion compared to ISTX and LSTX (P < 0.001 and P < 0.001, respectively). The ratio of percutaneous coronary intervention was higher in patients with LSTX and the ratio of CABG surgery was higher in patients with HSTX.

**Table 3:** Angiographic assessment and therapy choice by SYNTAX score groups.

	LSTX	ISTX	HSTX	P-value
Multivessel disease, n (%)	130 (51.0)	84 (82.4)	33 (100)	<0.001
Chronic total occlusion, n (%)	27 (10.6)	35 (34.3)	22 (66.7)	<0.001
Type of procedure, n (%) MedicalPCICABG	3 (1.2)241 (94.5)11 (4.3)	2 (1.9)80 (77.7)21 (20.4)	0 (0.0)15 (45.5)18 (54.5)	<0.001

PCI, Percutaneous coronary intervention; CABG, coronary artery bypass grafting.

**Figure 2:**
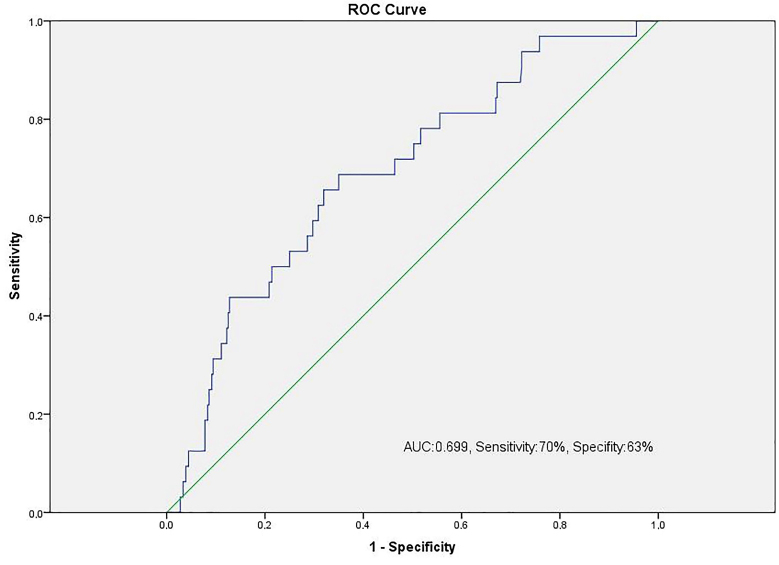
The receiver-operating characteristic (ROC) curve analysis for N-terminal probrain natriuretic peptide (NT-proBNP) levels in predicting patients with HSTX. Area under the curve (AUC): 0.699 (0.606–0.791).

**Figure 3:**
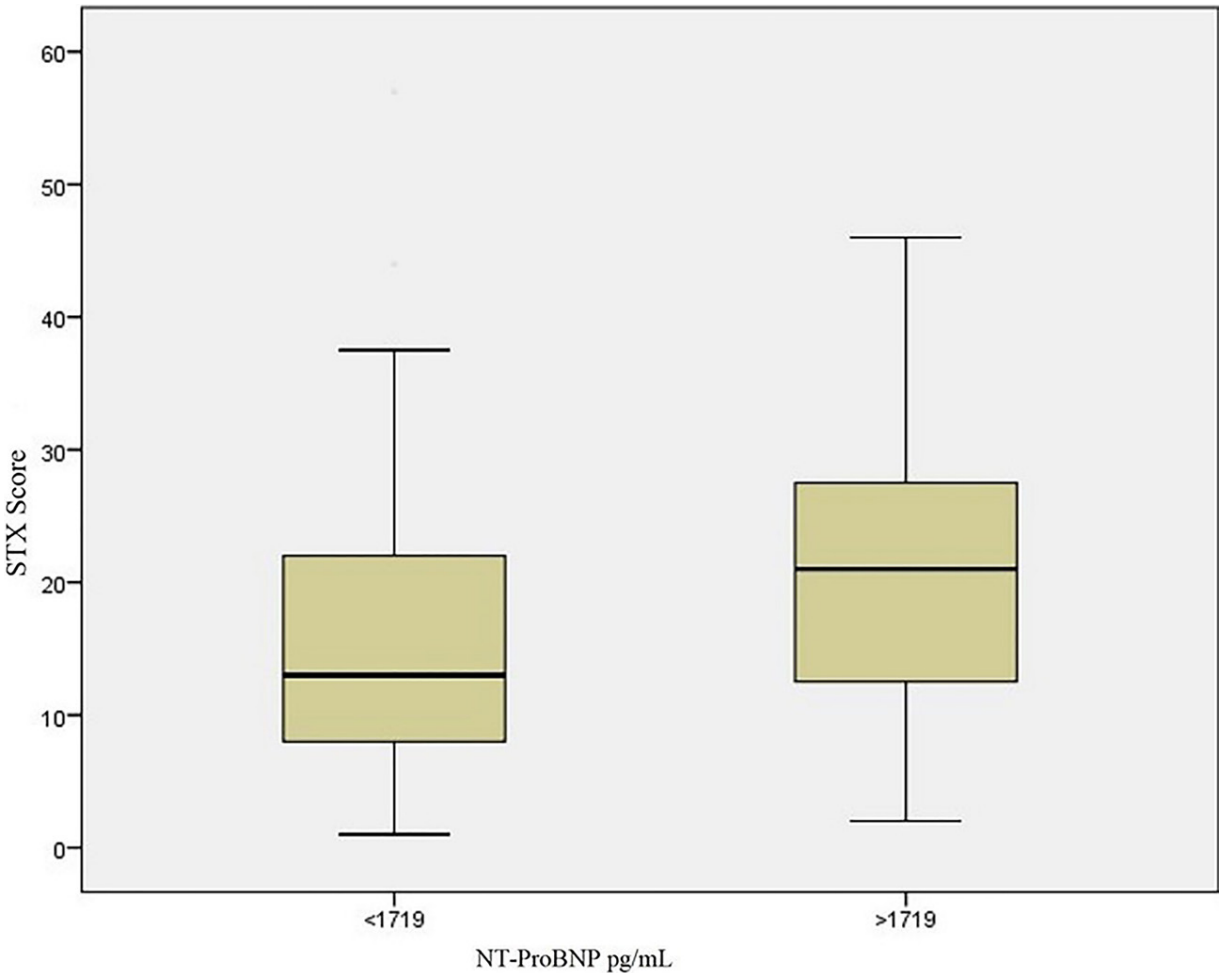
Patients were divided into two groups based on the cut-off point of 1719 pg/mL; mean STX score was significantly higher in the patients who had NT-proBNP levels above this cut-off value.

The optimal value of the cut-off point for NT-proBNP to predict HSTX was 1719 pg/mL (sensitivity 70%, specificity 63%) (Figure 2). The patients were divided into two groups based on the cut-off point of 1719 pg/mL; mean STX score was significantly higher in the patients who had NT-proBNP levels above this cut-off value (Figure 3).

Univariate logistic regression analyses were used to determine independent predictors of HSTX (variables: age, diabetes mellitus, smoking, family history, LVEF, WBC, glucose, creatinine, and NT-proBNP [>1719 pg/mL]). Multivariate logistic regression analyses were performed for significantly obtained variables (age, diabetes mellitus, LVEF, glucose, and NT-proBNP [>1719 pg/mL]). After multivariate logistic regression analysis we found that the strongest predictors of HSTX were increased serum NT-proBNP (>1719 pg/mL) (OR: 2.982, 95% CI: 1.328–6.694, P = 0.008), age (OR: 1.041, 95% CI: 1.012–1.073, P = 0.004), and serum glucose level (OR: 1.004, 95% CI: 1.001–1.008, P = 0.024) (Table 4).

**Table 4:** Predictors of high SYNTAX score (>33) in univariate and multivariate analyses.

	Univariate OR (95% CI)	P-value	Multivariate OR (95% CI)	P-value
Age	1.055 (1.025–1.087)	<0.001	1.041 (1.012–1.073)	0.004
Diabetes mellitus	3.553 (1.679–7.517)	0.001	1.840 (0.884–5.032)	0.056
Smoking	0.479 (0.216–1.065)	0.071		
Family history	0.147 (0.020–1.098)	0.062		
LVEF	0.942 (0.906–0.979)	0.003	0.961 (0.921–1.003)	0.070
WBC	0.888 (0.786–1.002)	0.055		
Glucose	1.005 (1.002–1.009)	0.004	1.004 (1.001–1.008)	0.024
Creatinine	2.853 (0.945–8.610)	0.063		
NT-proBNP >1719 pg/mL	3.643 (1.722–7.708)	0.001	2.982 (1.328–6.694)	0.008

LVEF, Left ventricle ejection fraction; WBC, white blood cell count; NT-proBNP, N-terminal probrain natriuretic peptide.

## 4. Discussion

In the present study we found that high NT-proBNP levels were associated with high SYNTAX scores in patients with STEMI and NSTEMI. NT-proBNP levels were found to be significantly higher in the HSTX group compared to the other groups, and in the ISTX group compared to LSTX group. The NT-proBNP levels demonstrated an increase from low STX score to high STX score tertiles. Thus, in the present study, we suggest that the NT-proBNP level is related with severity and complexity of CAD in patients with acute MI.

It has been shown that cardiomyocyte tension leads to BNP secretion [17,18]. BNP secretion increases when myocardial wall tension increases [19]. Elevated BNP levels were observed in systolic and diastolic heart failure, which were the cause of increased end-diastolic pressure [20–22]. Increased NT-proBNP is associated with severity of ischemia [23,24] and poor prognosis in ACS [25,26]. 

The SYNTAX score is used to detect the extent and complexity of CAD [1,27] and to predict prognosis in patients with CAD [3–5]. Akgün et al. demonstrated that STX is an independent predictor of both in-hospital and long-term mortality and major adverse cardiac events in patients with STEMI undergoing primary percutaneous coronary intervention (PCI) [28]. In the angiographic substudy of the ACUITY study of 2627 patients with NSTEMI undergoing PCI, STX was an independent predictor of 1-year rates of mortality, cardiac death, MI, and target vessel revascularization [29]. Valgimigli et al. demonstrated that STX predicts clinical outcome in patients with three vessel lumen obstruction undergoing PCI [30]. 

Previous studies have shown a correlation between STX and some biochemical parameters such as hs-troponin, LDL, ApoB, procalcitonin, and hs-CRP [6–8,10]. In the present study, we found that high NT-proBNP levels were associated with HSTX in patients with acute MI. The most likely cause of this result is increased wall stress caused by MI. Early studies showed that acute ischaemia increases left ventricular end diastolic pressure [31] and the main trigger mechanism for BNP secretion is cardiomyocyte tension [32]. Ischemia itself, on the other hand, leads to BNP release [33]. Another mechanism may be disruption of cell wall integrity during ischemia that leads to BNP release independently of hemodynamic effects [34]. Neurohumoral factors secreted during MI and tachycardia also lead to BNP release [35,36]. Thus, these mechanisms could contribute to NT-BNP release. Patients with high STX scores have greater ischemic burden, more wall tension elevation, and more necrosis; it is to be expected that these patients have higher NT-proBNP levels.

Early risk stratification is very important in patients presenting with suspected acute myocardial infarction due to major clinical relevance for identifying individuals at risk of death and guiding further diagnostics and/or therapeutic pathways. Different risk stratification scores are used in clinical practice, such as the TIMI score, GRACE score, log EUROSCORE, AMIS score, and others. These scoring systems use some clinical and laboratory parameters. We suggest that NT-proBNP could be used as a laboratory parameter for early risk stratification in patients with myocardial infarction.

There was a strong relationship between NT-proBNP levels and STX tertiles in this study. NT-proBNP levels gradually rose from the LSTX to the HSTX group. We found that NT-proBNP (>1719 pg/mL), age, diabetes mellitus, LVEF, and glucose were independent predictors of HSTX and increased serum levels of NT-proBNP were a strong predictor of HSTX in patients with MI. Multivessel disease, chronic total occlusion, and CABG were significantly higher in the HSTX group than in the other groups. Patients who have greater ischemic burden have higher NT-ProBNP levels. The relationship between NT-proBNP and poor prognosis is not completely explained. We suggest that high STX scores may explain this relation.

In conclusion, NT-ProBNP levels in patients with STEMI and NSTEMI on admission were independently associated with the extent, severity, and complexity of coronary atherosclerosis as assessed by STX score. NT-proBNP levels can be used for early risk classification in clinical practice in patients with MI.

This study has several limitations. First, STX was calculated from coronary angiographic findings by visual interpretation. Second, the concentration of NT-proBNP was only measured at admission and without correction for potential variability.
